# Hysteria as Seen at a Base Hospital

**Published:** 1919

**Authors:** 


**Hysteria as Seen at a Base Hospital.** By J. L. M. Symns. *The Practitioner*, August, 1918.

Babinski showed that hysteria should be considered as a definite pathological condition, caused by suggestion, which can be cured by suggestion. Hysteria, as we recognize it, is the subconscious representation of the patient's idea of the disease from which he considers himself to be suffering. The functional paraplegic who has been buried, will portray, subconsciously, the symptoms that he expects to find in a patient whose spinal cord was injured, and the greater his knowledge the nearer he will get to the signs of an organic paralysis. If this is the case, how is it that Charcot and his followers have found their stigmata present in the majority of their cases? Hemianesthesia, pharyngeal anesthesia, concentric narrowing of the visual field, and the various painful areas, such as the iliac fossa, are none of them symptoms which are likely to suggest themselves to the mind of the ordinary patient.

The author proposes to analyze these stigmata, and show why they have been found by the followers of Charcot, and not by those of Babinski.

*Pharyngeal Anesthesia.* The fact that pharyngeal anesthesia was frequently found in hysteria was noted by Charcot, while the fact that it is often absent in normal people, was not.

*Hemianesthesia.* This class of stigmata is the result of unconscious suggestion by the medical officers, and we ourselves have no difficulty in producing it by suggestion.

*Visual Stigmata.* We have examined the vision of all cases by the rough finger method, which involves no suggestion, and have found the field of vision normal.

*Hysterogenic Zones.* We have never found the tender point over the anatomical site of the ovary to be indicative of hysteria, although Purves Stewart quotes Steinhausen as finding it in 88 % of 500 normal soldiers.

The observation that hysteria does not arise in the field is borne out by the statements of medical officers, as quoted by Babinski and Froment.

Hysteria is caused by suggestion. With the rest and reaction after the strain of action, the patient has the time and opportunity to think and brood over his condition, and then the hysterical symptoms appear.

The author proposes to note the chief types met with :

1. *Hysterical Gaits.* (*a*) The tacking gait, in which the patient is unable to keep to a straight line and tacks from side to side. (*b*) The pseudo-ataxic gait, which varies from a mere difficulty in walking to an absolute loss of equilibrium. (*c*) The pseudo-spastic gait, in which arms and legs and body are held rigid. (*d*) The unilateral gait, in which the patient moves with a lateral swaying movement. (*e*) The senile gait, in which the back is bent and the patient walks with two sticks.

2. *Hysterical Paralysis.* (*a*) Pseudo-hemiplegia. (*b*) Pseudo-paraplegia. (*c*) Pseudo-monoplegia. (*d*) Pseudo-paralysis of an anatomical region or a group of muscles.

3. *Hysterical Contractures.* (*a*) Hysterical club foot, in which the foot is immovable and extended, and the patient walks on the external border of the foot. (*b*) Hysterical contractures of the hand, resulting in the typical " main d'accoucheur ", or, in other cases, in the flexion of one or more fingers. (*c*) Hysterical contracture of muscles.

4. *Hysterical Tremors.* (*a*) Generalized atypical tremors. (*b*) Limited atypical tremors. (*c*) Typical tremors, which are similar to those found in disseminated sclerosis or paralysis agitans.

5. *Hysterical Errors of Speech.* (*a*) Dumbness. (*b*) Peculiarities of speech : (1) Stammering. (2) Latent period between words. (3) Inarticulate. (4) Whispering. (5) Associated movement of limb.

6. *Hysterical Fits.* (*a*) Emotional, rarely seen in our experience but common among the French, who describe them under the term " grande hystérie ". (*b*) Pseudo-epileptic, which represent the patient's idea of a fit, and are associated with fear.

7. *Hysterical Deafness.* Usually follows upon the inability to hear during a prolonged bombardment.

8. *Visual Disorders.* Complete amaurosis, photophobia and blepharospasm are chiefly seen.

9. *Alimentary Hysteria.* (*a*) Vomiting. (*b*) Constipation.

10. *Respiratory Hysteria.* Tachypnea. In these cases there is sudden acceleration of the respiratory movements, usually associated with a marked emotional state.

11. *Disorders of Sphincter-Control.* This class of patients gives no previous history of incontinence, and responds to treatment by reeducation and suggestion.

*Diagnosis.* In making the diagnosis we have to rely on the absence of those objective signs which characterize diseases in the nervous system : (*a*) Optic neuritis, optic atrophy, heminanopia; (*b*) alterations in electrical reactions of muscles and nerves; (*c*) alterations in the superficial reflexes, e. g., extensor plantar reflex; (*d*) loss or great exaggeration or inequality in the deep reflexes, such as knee and ankle jerks.

There are also other signs of an organic lesion which are of considerable importance :

(*a*) Babinski's second sign. Combined flexion of the thigh and trunk on the paralyzed side when the patient attempts to rise from the prone to the sitting position, the arms being folded.

(*b*) The pronation sign, in which the hand when tossed assumes a position of pronation.

(*c*) The fan sign. Spreading of the toes associated with the extensor, plantar response, obtained when the sole of the foot is stimulated.

(*d*) The presence of hypotonus, shown by the exaggerated flexion of the forearm on the affected side.

(*e*) The platysma sign, which is shown by the absence or diminished movements of the fibers of the platysma on the affected side when the mouth is open.

The great factor in the cure is an atmosphere of cure. The patient is placed with others who are now well, and one can always count on their telling him of their recovery. According to the degree of the hysterical lesion, direct suggestion, continuous suggestion, hypnosis, ether, or intralaryngeal catheterization are used.

The treatment, as the author has pointed out, depends largely on suggestion, and it is extremely difficult to describe the routine carried out in each case.

The great secret of the treatment lies in the seizing of any signs made by the patient that will serve to suggest a cure, and following it up until the hysterical lesion is mastered. It is always difficult to say how long the treatment will take. Hysteria is a pathological condition caused by suggestion and cured by suggestion.

